# Considerations for Size, Surface Charge, Polymer Degradation, Co‐Delivery, and Manufacturability in the Development of Polymeric Particle Vaccines for Infectious Diseases

**DOI:** 10.1002/anbr.202000041

**Published:** 2021-01-18

**Authors:** Christopher J. Genito, Cole J. Batty, Eric M. Bachelder, Kristy M. Ainslie

**Affiliations:** ^1^ Department of Microbiology and Immunology University of North Carolina at Chapel Hill 4211 Marsico Hall, 125 Mason Farm Road Chapel Hill NC 27599 USA; ^2^ Division of Pharma Engineering & Molecular Pharmaceutics Eshelman School of Pharmacy University of North Carolina at Chapel Hill 4211 Marsico Hall, 125 Mason Farm Road Chapel Hill NC 27599 USA

**Keywords:** controlled release, manufacturing, nanoparticles, polymeric particles, vaccines

## Abstract

Vaccines have advanced human health for centuries. To improve upon the efficacy of subunit vaccines they have been formulated into nano/microparticles for infectious diseases. Much progress in the field of polymeric particles for vaccine formulation has been made since the push for a tetanus vaccine in the 1990s. Modulation of particle properties such as size, surface charge, degradation rate, and the co‐delivery of antigen and adjuvant has been used. This review focuses on advances in the understanding of how these properties influence immune responses to injectable polymeric particle vaccines. Consideration is also given to how endotoxin, route of administration, and other factors influence conclusions that can be made. Current manufacturing techniques involved in preserving vaccine efficacy and scale‐up are discussed, as well as those for progressing polymeric particle vaccines toward commercialization. Consideration of all these factors should aid the continued development of efficacious and marketable polymeric particle vaccines.

## Graphical abstract

Properties such as size, surface charge, degradation rate, and the co‐delivery of antigen and adjuvant all affect the immune responses of polymeric particle vaccines for infectious disease. A review of current and past literature reveals these effects vary with polymer type, antigen, adjuvant, and route of administration. Vaccine manufacturing methods also impact efficacy, scalability, and marketability.

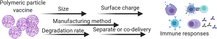

## 1 Introduction

Vaccines can easily be identified as one the most influential medical advances for human health, among the similarly important advances of sanitation, nutrition, and antibiotics.^[^ ^1^ ^]^ Edward Jenner is credited with the modern idea of vaccination, based on his documented evidence in the late 1700s that cowpox inoculation could prevent smallpox infection.^[^ ^2^ ^]^ However, the idea that previous exposure to infection can prevent disease dates as early as 430 BC. Athenian nurses who recovered from a plague similar to smallpox were thought to be resistant to reinfection and were used to attend to new patients.^[^ ^3^ ^]^ Later reports have cited Chinese physicians inoculating with smallpox pustules (variolation) intranasally in the 10th century and variolation may have been practiced in China as early as the 8th century, reaching Europe much later.^[^ ^4^, ^5^, ^6^ ^]^ Indeed, the well‐traveled Lady Mary Wortley Montagu inoculated her children in England with pustules in 1716.^[^ ^6^, ^7^ ^]^ From there, the controversial idea of inoculation with pustules was practiced among the educated, with Jenner himself being vaccinated at age eight.^[^ ^7^ ^]^ However, it was Jenner who coined the term “vaccine” (from the Latin *vacca*, meaning “cow”) based on his isolation of cowpox from cows.^[^ ^8^ ^]^

Jenner's use of a live cowpox virus, which had an attenuated response in humans with respect to the pathogenic smallpox virus, is a classic example of an attenuated pathogen vaccine. An example of a similar practice still used in the clinic today is the Bacille Calmette–Guerin (BCG) vaccine. Derived from *Mycobacterium bovis* (the causative agent of bovine tuberculosis), the BCG vaccine provides protection, albeit variable and limited, against *Mycobacterium tuberculosis* (Mtb) infection in humans. Other commonly used attenuated vaccines include the measles, mumps, and rubella (MMR) vaccines and the intranasal influenza FluMist vaccine. As historically common as this type of vaccine may be, attenuated vaccines carry concerns related to potential infection in individuals with a suppressed immune system (e.g., HIV+, transplant, or cancer patients). An example of these adverse effects can be seen with the oral polio vaccine. Composed of a polio virus attenuated to reduce its invasion into nerves, this vaccine can nonetheless induce paralysis in immunocompromised patients, and upon mutation become a more virulent strain of the virus.^[^ ^9^, ^10^ ^]^ In addition, the method of attenuation can result in decreased efficacy, such as what was observed with FluMist and resulted in the vaccine being pulled from the market for several years.^[^ ^11^ ^]^

Another common type of vaccine involves the use of inactivated pathogens, illustrated by Louis Pasteur's work with cholera and anthrax,^[^ ^12^ ^]^ and Jonas Salk's work with polio.^[^ ^13^ ^]^ Clinically, the most commonly used intramuscular (IM) influenza vaccines are an inactivated viral vaccine, wherein virus is grown up in fertilized eggs, inactivated with formaldehyde or another similar agent, and purified for formulation, packaging, and distribution. Inactivated vaccines are considered safer than attenuated ones; however, they tend to be less immunogenic and require more boosts after the initial injection to provide protective immunity.

As vaccine development has evolved, identification of immunodominant and protective antigens (PAs) has resulted in the generation of subunit vaccines. The first Food and Drug Administration (FDA)‐approved subunit vaccine was developed by Hilleman and co‐workers at Merck in 1976 for hepatitis B.^[^ ^14^ ^]^ Subunit vaccines can be comprised of proteins, glycoproteins, or polysaccharides from the pathogen. As subunit vaccines are only elements of the pathogen, they are considered the safest type of vaccine; however, they are often poorly immunogenic, and it is thought they may lead to the development of vaccine resistance.^[^ ^15^ ^]^ Adjuvants are often required to develop efficacious subunit vaccines, however these adjuvants serve as immunostimulants that can add additional safety concerns. One example is in the comparison of two virus‐like particle (VLP) subunit vaccines, Cervarix and Gardasil. It was observed that Gardasil had reduced efficacy compared to Cervarix, even though the two vaccines contained similar human papillomavirus proteins.^[^ ^16^ ^]^ Although both formulations include aluminum salts (alum), Cervarix additionally contains 3‐O‐desacyl‐4′‐monophosphoryl lipid A (MPL). This formulation of MPL and alum is part of the GlaxoSmithKline (GSK) adjuvant system (AS) of adjuvants and is termed AS04. AS04 enhances the immunogenicity of the Cervarix VLP and is likely responsible for the vaccine's increased efficacy. However, evidence of possibly increased adverse effects led to Cervarix being removed from the US market due to low demand.^[^ ^17^, ^18^ ^]^

Closely related to subunit vaccines are the rapidly advancing nucleic acid vaccines. As the name implies, these vaccines use nucleic acids such as mRNA or plasmid DNA to encode the antigen(s). Rather than administering antigen directly as with a conventional subunit vaccine, nucleic acid vaccines use the host cell machinery to produce the encoded antigen. Vaccines based on nucleic acid were first identified in the early 1990s when plasmid was delivered intramuscularly and a humoral response was observed.^[^ ^19^ ^]^ mRNA‐based vaccines were discovered shortly thereafter.^[^ ^20^ ^]^ Although DNA‐based vaccines require nuclear delivery, mRNA vaccines only need to be delivered to the cytoplasm. In addition to the added delivery barrier for DNA vaccines, there is an added safety concern that the plasmid could in theory incorporate in the host genome, although this has not been reported.^[^ ^20^ ^]^ Delivery of nucleic acid vaccines can be improved by incorporation into particle delivery systems to facilitate cellular entry and protect the cargo from nuclease degradation.^[^ ^21^ ^]^ Polymeric encapsulation or complexation has been used to deliver mRNA and other nucleic acid vaccines,^[^ ^22^ ^]^ but complexing it to cationic and ionizable lipids is much more common. Indeed, the two leading vaccine candidates for the COVID‐19 pandemic, developed by Moderna and Pfizer/BioNTech, are a lipid nanoparticle (NP) that complexes mRNA encoding the SARS‐CoV‐2 spike protein.^[^ ^23^, ^24^, ^25^ ^]^ If approved, COVID‐19‐based vaccines would be the first nucleic acid vaccines to be approved by the FDA. At the time of writing this review, Pfizer/BioNTech has been approved for application in the United Kingdom^[^ ^26^ ^]^ and Moderna has filed for emergency FDA approval of their vaccine.^[^ ^27^ ^]^

Concerns of vaccine safety, both legitimate and perceived, have led to anti‐vaccine sentiments over the course of several centuries.^[^ ^28^ ^]^ To help mitigate the concern of safety, the formulation of vaccine antigens and adjuvants into biomaterial carriers, such as nano/microparticles (NPs), has been an increasing area of study since 1976 when liposomes^[^ ^29^ ^]^ and polymeric (poly(methyl methacrylate) (PMMA))^[^ ^30^ ^]^ were first reported as vaccine carriers. Since their emergence in the 1970s, a handful of NP vaccines have made it to clinical approval. Internationally approved, Epaxal and Inflexal V are inactivated hepatitis A and influenza viruses, respectively, that are encapsulated in a liposome.^[^ ^31^, ^32^, ^33^ ^]^ Inflexal V can be administered to a wider range of ages than some flu vaccines, indicating more stable immunogenicity and safety profiles.^[^ ^33^, ^34^ ^]^ In addition to encapsulating inactivated viruses, liposomes have been used to deliver adjuvants. The FDA‐approved shingles vaccine Shingrix (approved 2017) and internationally‐approved malaria vaccine RTS,S/AS01 (EU‐approved 2015) are adjuvanted with AS01, which is a liposomal formulation of the adjuvants MPL and QS‐21. Of the two shingles vaccines available domestically in the US, the Shingrix glycoprotein subunit vaccine outperforms the live‐attenuated Zostavax for efficacy and longevity, with similar observed safety.^[^ ^35^ ^]^ Although a clear potential for particulate vaccines has been shown, only a handful of liposomal vaccines and no polymeric particle vaccines have made it to FDA approval.

Polymeric particle vaccines represent a new approach to improve upon existing formulation strategies. Building on work by Sandoz (now part of Novartis) in 1976 investigating antigens adsorbed on PMMA NPs,^[^ ^30^, ^34^ ^]^ the first synthetic biodegradable (poly(lactide‐*co*‐glycolide) or PLGA) NPs were reported in 1991^[^ ^36^, ^37^, ^38^ ^]^ as controlled‐release systems for vaccines. A slow (controlled) release of vaccine elements, through what is known as a “depot effect”, is thought to be one of the potential mechanisms of the ubiquitously used adjuvant alum, which has been used clinically since the 1920s.^[^ ^39^, ^40^ ^]^ In addition, the idea of a vaccine depot dates back to at least 1944^[^ ^41^, ^42^ ^]^ with Freund's adjuvant (now Complete Freund's adjuvant or CFA), which uses an oil–water emulsion with inactivated and dried Mtb. By suspending the antigen in an oil and water mixture, the residence time of the antigen increases, with the prospect of continuously stimulating antigen‐presenting cells (APCs) and improving adaptive immune responses to the antigen. Several FDA‐approved adjuvants also use emulsions (without the addition of Mtb) for the controlled release of vaccine elements, including MF59 and AS03, which are used for seasonal and pandemic flu vaccines, respectively. These emulsions primarily consist of shark oil (squalene), water, and an amphiphilic molecule (e.g., polysorbate 80, span 85). However, these squalene‐based systems carry potential safety concerns, including reports of narcolepsy in children.^[^ ^43^, ^44^, ^45^ ^]^ Though narcolepsy events associated with AS03 may also be linked to the influenza nucleoprotein,^[^ ^46^ ^]^ isolated events of increased anaphylaxis have also been reported with AS03.^[^ ^47^, ^48^ ^]^ There have been isolated reports of deaths thought to be linked to FluAd, which contains MF59, in Italy where the vaccine was approved in 1997.^[^ ^49^, ^50^ ^]^ FluAd was approved by the FDA in 2015. Another adjuvant, AS02, also uses a squalene‐based system to suspend liposomal QS‐21 and MPL. AS02 has been previously used in clinical trials as part of a malaria vaccine (RTS,S/AS02), but was discontinued in favor of the AS01 adjuvant which does not include squalene.^[^ ^51^ ^]^ These adjuvants illustrate the idea that a controlled release or depot system for a vaccine can effectively be brought to the clinic. However, safety concerns (e.g., injection site reactions, vascular leak syndrome, acute phase response resulting in fever and other flu‐like symptoms)^[^ ^52^ ^]^ provide motivation for a new approach to achieve sustained release, such as the use of safe biodegradable polymeric particles.

Although a polymeric particle vaccine has yet to achieve FDA‐approval, much work has been done in the past several decades to advance polymeric NP‐based vaccine formulations. Continuing on the work first reported in 1991 by multiple groups, the WHO supported PLGA NPs for tetanus vaccination in children, but ultimately failed in implementing the vaccine.^[^ ^53^ ^]^ The vaccine was not successful for several reasons including denaturation of the antigen due to the low pH generated internally by the polymer's degradation, that alum was still required, and that it was cost‐prohibitive to make particles on an industrial scale for application in a resource‐limited setting.^[^ ^54^, ^55^, ^56^ ^]^ Since this advance, there has been continued work on PLGA and other biodegradable polymeric vaccine NP formulations. This work has led to a better understanding of how altering various physical properties of NPs, including particle size, surface charge, degradation rate, and the co‐encapsulation of vaccine elements, can impact the resulting immune response and the effectiveness of the vaccine. Some of these particle formulations can preclude the use of alum or other vaccine adjuvants, and better manufacturing methods have led to lower production costs and increased feasibility for NP‐based vaccines. We review here these advances in polymeric particle vaccine formulations which have led to preclinical results that can advance the technology beyond the limitations of the initial tetanus work.

## 2 Considerations for Comparing Vaccines

### 2.1 Routes of Vaccination

Countless particulate vaccine systems have been developed over the decades, and the physiochemical properties of many of these systems have been reported and reviewed.^[^ ^57^, ^58^, ^59^, ^60^, ^61^, ^62^, ^63^, ^64^, ^65^, ^66^, ^67^ ^]^ A number of these reviews compare both mucosal (e.g., intranasal, oral) and injectable (e.g., IM, subcutaneous [SC]) vaccines. However, the immune responses resulting from mucosal and injectable vaccines are exceedingly different.^[^ ^8^, ^68^ ^]^ Considering the antibody response, the goal of most mucosal vaccines is to elicit antibody responses in the mucosal spaces, of which the IgA class of antibodies is a major component. Injectable vaccines are often measured by serum antibody levels, where IgG predominates. Although mucosal vaccines can elicit serum antibodies, serum IgG titers do not represent the goal or the totality of the immune response for these vaccines. In addition, the adjuvants for mucosal responses are drastically different than injectable vaccines. For example, alum is the most used adjuvant in injectable subunit vaccines, but poorly induces mucosal immune responses.^[^ ^69^ ^]^ Major considerations in the design of particulate vaccines for mucosal delivery are also distinct from injectable vaccines. While physical particle properties such as charge and the polymer used can influence the immune response to injectable vaccines, these properties can also influence mucoadhesion and the interface of mucosal vaccines with mucosal‐associated lymphoid tissue.^[^ ^70^ ^]^ Thus, conclusions made about how particle physical properties affect the immune response can be drastically different between mucosal and injectable vaccines. For these reasons, discussions here focus on injectable vaccines. Considerations for polymeric particle vaccines for mucosal applications have been discussed in multiple recent reviews.^[^ ^70^, ^71^, ^72^ ^]^

As an additional consideration, the intradermal (ID) route offers unique trafficking and engulfment compared with other injectable routes. For example, ID injection has an increased residence time compared with IM, SC, or intraperitoneal (IP) routes because the dermal space is more hydrophobic than the interstitial space and tissue into which the other routes are injected. This often translates to a reduction in the required antigen amount for ID formulations, as can be seen with the influenza vaccine Fluzone which has both an IM and ID formulation. The ID formulation contains only 15% of the antigen dose of the IM formulation, as well as 20% the total volume (product insert). Though the volume that can be injected ID is smaller, the persistence of vaccine elements is longer than other routes through an enhanced depot effect. Therefore, to make direct comparisons, this review focuses on needled‐delivery of vaccines through IM, SC, and IP routes.

### 2.2 Evaluation of Endotoxin

One physiochemical property of vaccines that is often not reported in preclinical work is the evaluation of endotoxin. Endotoxin is comprised of lipopolysaccharides (LPSs) which stimulate toll‐like receptor (TLR) 4 and cannot be removed via sterile filtering (0.2 μm). The presence of endotoxin can significantly alter innate signaling both in vitro and in vivo. Endotoxin evaluation is often reported in more recent publications, but past work may require reevaluation of any endotoxin‐related innate signaling that may have significantly influenced the immune response. This is especially true for treatments including antigen, as some types of low‐grade ovalbumin, or bacterial‐sourced recombinant proteins contain substantial amounts of endotoxin. It would also be prudent to rule out endotoxin contamination as an explanation for blank vehicle treatments that elicit an innate immune response above background. Polymer, solutions used to make particles, or solutions in devices used to make particles can be purchased endotoxin‐free; however, these reagents could develop endotoxin contamination due to bacterial growth if exposed to an unsterile environment and stored at ambient temperature for long periods of time. Although previously endotoxin detection required a specialized machine for solely that function, endotoxin detection via limulus amebocyte lysate (LAL) measurement can now be performed using a commercially available colorimetric assay and a plate reader. With the increased access to endotoxin detection, it will no doubt become more common in future peer‐reviewed publications. For this review, we have indicated if endotoxin was evaluated in the reported primary literature with the presence of an asterisk (*).

## 3 Effect of Physical Properties on Vaccine Responses

### 3.1 Particle Size

Physiochemical properties such as size, charge, and shape can significantly affect three main aspects of particle interfacing with the immune system: 1) Trafficking of particles and/or payload to antigen presenting cells (APCs) and lymph nodes (LNs). 2) Engulfment of particles by APCs or the direct activation of B cells. 3) Phagosomal trafficking of particles/payload within the cell. Factors such as peptide presentation, pattern recognition receptor (PRR) activation, cell specific uptake, or major histocompatibility complex (MHC) cross‐presentation are all dependent on one or more of these three processes. Moreover, these three processes dictate the measured outcomes of innate, cellular, memory, and protective responses. To this end, evaluation of size, shape, charge, and targeting with respect to these three elements sometimes reveal that the effects of these physiochemical features cannot be easily delineated from each other to truly identify the interdependence of each on immunological responses. To add to this list, the effect of polymer degradation on the immune response of particle‐based vaccines has been reported more recently and shown to exert significant effect on phagosomal trafficking and overall efficacy.^[^ ^73^, ^74^, ^75^ ^]^ With this in mind, nondegradable polymeric systems such as inorganic (e.g., gold, silicon) and polystyrene particles are difficult to compare to degradable polymer systems. In addition, common fabrication methods of polymeric particles often result in increased polydispersity compared with inorganic and polystyrene particles that are, in general, very monodisperse. Sharp et al. reported that when comparing PLGA microparticles (MPs) to polystyrene particles of different sizes, the two polymers invoked different innate signaling and inflammasome activity, indicating that either polydispersity, degradation, or another factor influenced immune signaling.^[^ ^76^ ^]^

**Figure 1:**
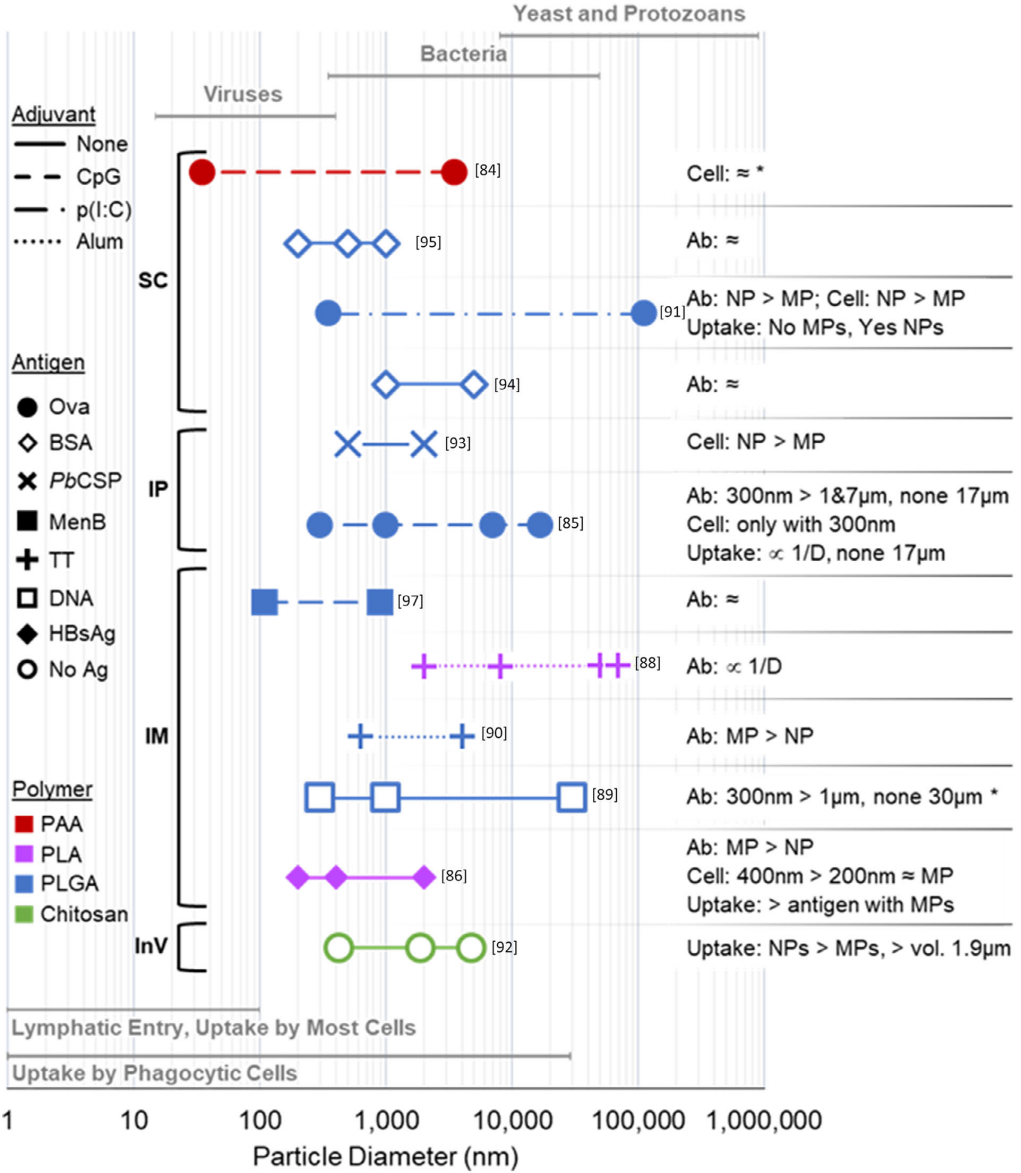
Summary of particle diameters studied for injectable polymeric particle vaccines. The range of surface size tested in each experiment is denoted by a line; points represent the average particle diameter (D) for each particle batch tested, as indicated on the *X*‐axis. The shape of each point denotes if no antigen (No Ag); a protein antigen including ovalbumin (Ova), bovine serum albumin (BSA), *Plasmodium berghei* circumsporozoite protein (*Pb*CSP), *Neisseria meningitides* type B antigen (MenB), TT, and hepatitis B surface antigen (HBsAg); or HIV‐1 p55 DNA was used. The line pattern denotes whether Alum, CpG, Poly(I:C), or no adjuvant was used. The color of each point/line reflects the polymer used (PAA = polyacrylamide). Route of particle administration is denoted to the left of each line (SC = subcutaneous, IP = intraperitoneal, IM = intramuscular); “InV” indicates the experiment was performed in vitro. Effects on the immune system are summarized to the right of each line, with “Ab” meaning antibody response, “Cell” referring to generalized cellular response, and “Uptake” referring to cell uptake. “∝” means “proportional to” and “≈” means approximately equal and not statistically different. When not indicated, conclusive data was not presented on that topic. If a range of sizes are reported in the publication, then the smallest size is presented. Sizes of pathogens listed at the top and route of particle trafficking at bottom in boxes are modified from Bachmann and Jennings.^[^ ^67^ ^]^ An asterisk (*) signifies that endotoxin testing of the final particle formulation was reported.

The immune system has adapted to combat pathogens that can range in size from 1 nanometer to 100 μm^[^ ^67^ ^]^ (**Figure** **1**). For both immunity induced by natural infection and vaccination, all or part of the pathogen or associated antigen is taken up by APCs such as dendritic cells (DCs) and macrophages for the stimulation of the adaptive immune response and to promote immune memory. It is known that bacterial cell uptake by APCs is dictated by factors mostly independent of size and shape, often due to the biochemical complexity and size variety of bacteria.^[^ ^77^, ^78^ ^]^ It was reported that biodistribution in vivo of injected particles with a hydrodynamic radius less than 100 nm can convect to lymph vessels and eventually be trafficked to LNs or the circulation.^[^ ^79^ ^]^ In contrast larger particles, up to the size of a macrophage (≈20 μm)^[^ ^80^, ^81^ ^]^ would require phagocytosis for trafficking to the LNs and will persist at the injection site until clearance by phagocytic cells (e.g., macrophages, DCs).^[^ ^82^ ^]^ Likely due to the ability to size particle systems within the range of pathogens, size has been highly studied for vaccine particle systems.

A summary of injectable and degradable particle systems used to evaluate size in the context of vaccines is presented in Figure 1. In this summary of work, very few studies coincide regarding similar polymers, sizes, and routes. The heterogeneity of this collection of data makes it difficult to glean a governing rule regarding vaccine particles; however, there are many rules that have been presented in several review articles regarding size. For example, one review by Slütter and Jiskoot states that “the most consistent result [across the literature] appears to be the advantage of NPs (smaller than 200 nm) over MPs (>1 μm) at priming cytotoxic (CD8+) T cells.”^[^ ^65^ ^]^ This conclusion was based on only one, albeit thorough, study by Mottram et al.^[^ ^83^ ^]^ using PS beads (20, 40, 49, 67, 93, 101, and 123 nm) with covalently bound ovalbumin administered ID to the foot pad of mice. Notably, particles of sizes <200 nm were not directly compared with larger, >1 μm particles in this study. In addition, as stated earlier, PS particles can elicit different immune responses from particles made from biodegradable polymers, and the ID route differs from other injectable routes. Together, this highlights that one universal conclusion for size‐related effects that encompasses all polymers, and every route of injection is not feasible. Figure 1 presents a schematic summary of 12 studies that have reported comparison of multiple sized particles using degradable polymers with 11 in vivo studies using injectable routes.

The idea that small NPs elicit a stronger cellular response is also supported by a review from Benne et al.^[^ ^62^ ^]^ However, Benne et al. note that the route of particle administration influences the type of DC subset to which the particles are delivered.^[^ ^62^ ^]^ Since DCs are highly involved in T‐cell and other related vaccine responses, it is further indicated that generalizations cannot be made across routes for cellular responses. Moreover, multiple reports conclude that smaller particle size does not always carry an advantage for inducing cellular responses when using biodegradable polymers. Cohen et al. compared 35 nm and 3.5 μm polyacrylamide particles and reported that cellular activation was not statistically different when CpG was used, although smaller NPs were more activating when no adjuvant was used.^[^ ^84^ ^]^ This is in contrast to Joshi et al.'s report that cellular activation was only observed with 300 nm PLGA particles and not 1, 7, or 17 μm particles.^[^ ^85^ ^]^ Kanchan and Panda reported that cellular activation was greater for 400 nm PLA particles than 200 nm particles, and 200 nm particles elicited a similar cellular response to 2–8 μm particles.^[^ ^86^ ^]^ It would therefore seem that the effect of NP size on cellular responses may vary with the polymer used.

Taking antibody responses into similar consideration, vast differences in particle size‐related effects are observed within the same route of administration (IM). Wendorf et al.^[^ ^87^ ^]^ reported that antibody titers were not statistically different when comparing 110 and 800 nm NPs, whereas Katare et al.^[^ ^88^ ^]^ and Singh et al.^[^ ^89^ ^]^ reported antibody responses were inversely proportional to diameter when using PLA and PLGA particles, respectively. However, an additional publication by Katare et al.^[^ ^90^ ^]^ concluded that MPs elicited better antibody titers than NPs, even though similar polymers (PLGA or PLA) and the same antigen (tetanus toxoid) were used in both publications.^[^ ^88^, ^90^ ^]^ Kanchan and Panda also reported PLA MPs elicited higher antibody titers than NPs.^[^ ^86^ ^]^ These four studies, which all involved IM injected particles, demonstrate that size effects can vary even when using the same polymer type and route of injection.

Taken together, a clear pattern regarding size and immune response are difficult to glean. For uptake, three studies indicate that smaller particles are better.^[^ ^86^, ^91^, ^92^ ^]^ Yet, one study reports that, despite the greater uptake of NPs, the slow release from MPs led to more uptake of antigen and optimal antibody titers.^[^ ^86^ ^]^ It should be noted that the uptake of antigen is the end goal of vaccine delivery and not necessarily the uptake of particles, which is underscored by the fact that an inverse relationship between particle diameter and immune responses is not consistently observed. For cellular responses, three reports using the same polymer (PLGA) conclude that smaller is better.^[^ ^85^, ^91^, ^93^ ^]^ Using other polymers, one study indicates there is no difference between sizes^[^ ^84^ ^]^ and one that intermediate size was optimal.^[^ ^86^ ^]^ Moreover, with antibody responses, three studies indicated no difference,^[^ ^87^, ^94^, ^95^ ^]^ four identified that smaller particles were better^[^ ^85^, ^88^, ^89^, ^91^ ^]^ and two concluded larger particles were best,^[^ ^86^, ^90^ ^]^ with all studies using the same or similar polymers, but different routes of injection, antigen, and adjuvant. Clearly, particle size‐related trends in the resulting vaccine response are dependent on multiple factors. Together, the data in Figure 1 would indicate that the effect that size has on each species of particle vaccine varies with the associated route of administration, antigen, and polymer, which is also directly and indirectly supported by other reviews of particle size.^[^ ^59^, ^62^, ^64^, ^96^ ^]^ Although it is clear that changes in size can lead to varying immune responses, there is not an observable trend to conclude the role of size in polymeric particle formulations. Each formulation therefore requires individual characterization.

### 3.2 Surface Charge

**Figure 2:**
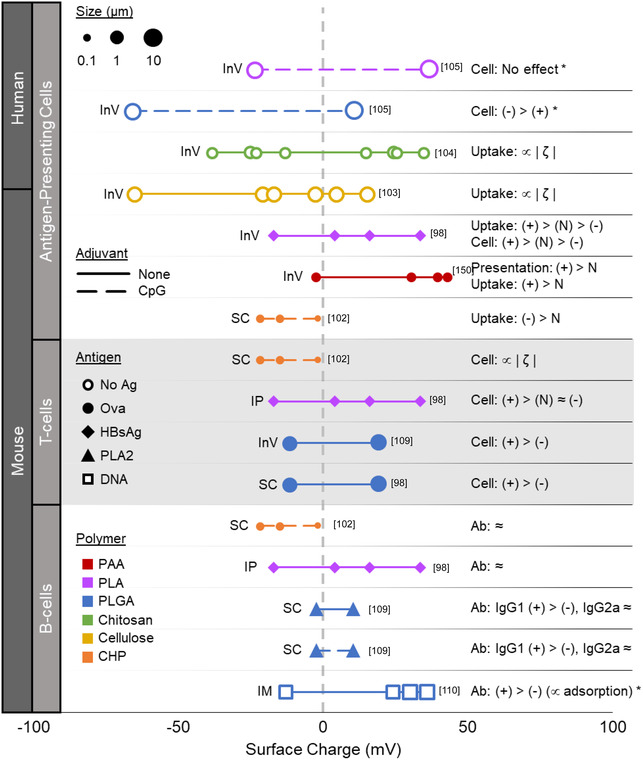
Summary of surface charge effects on immune response for injectable polymeric particle vaccines. Each point represents the average ζ‐potential/surface charge for each reported batch of polymeric particle at neutral pH. The size of the point is relative to the average particle diameter. For particle batches of similar size and charge, the batch with greater magnitude surface charge is shown. The shape of each point denotes if no antigen (No Ag), ovalbumin (Ova), HBsAg, Bee venom allergen PLA2, or HIV‐1 p55 DNA was used. The range of surface charge tested in each experiment is denoted by a line, which is dashed if an adjuvant (CpG) was used. The color of each point/line reflects the polymer used (PAA = polyacrylamide, CHP = cholesterol‐bearing pullulan). Route of particle administration is indicated to the left of each line (SC = subcutaneous, IP = intraperitoneal, IM = intramuscular); “InV” denotes experiment was performed in vitro. Charge effects on cell uptake (Uptake), generalized cellular responses (Cell) or antibody responses (Ab) are summarized to the right of each line, for cationic (+), anionic (−), or neutral (N) particles (≈ = minimal or not statistically significant differences, ∝ = “proportional to”, | ζ | = charge magnitude). An asterisk (*) signifies that endotoxin testing of the final particle formulation was reported.

Polymeric particle systems with a variety of surface charges, both positively and negatively charged, have been designed for use as vaccines. The average surface charge of particles is usually measured by zeta potential, with an absolute magnitude less than 100 mV. One method to intentionally impart a surface charge is to include charged polymers, such as poly‐l‐lysine (PLL) or polyethylenimine (PEI), in or on the polymeric NP. These charged polymers can be blended with neutral polymers or incorporated into co‐polymers to form charged NPs. The magnitude and sign of the charge varies with the concentration of the charged polymer with respect to the other polymers in the NPs (e.g., PLGA, PLA). Some polymers, such as chitosan, carry an inherent charge at neutral pH and can be used alone to construct charged particles. A main advantage of charged particles is the electrostatic interactions between the particle surface and other vaccine components, specifically the antigen and adjuvant, which can promote adsorption. In some cases, adsorption through electrostatic interactions can preserve the structure and function of protein antigens better than encapsulation.^[^ ^97^ ^]^ Another potential advantage of imbuing a surface charge on injectable polymeric particle vaccines is a greater immune response, which has been studied both in vitro and in vivo (**Figure** **2**).

In general, cationic particles show greater uptake and activation of APCs, namely macrophages and DCs, than anionic particles.^[^ ^98^, ^99^, ^100^, ^101^, ^102^ ^]^ However, this generalization comes with multiple caveats. First, multiple studies have shown that a greater magnitude of surface charge increases the effect, and that a sufficiently large negative charge on particles can increase uptake by APCs and subsequent activation when compared to particles with neutral charge.^[^ ^103^, ^104^, ^105^ ^]^ Second, anionic particles and cationic particles may differ in the way they interact with APCs other than increases in uptake and response. For example, cationic polystyrene particles formed through cationic surface modification were associated with decreased phagosome acidification compared with more neutral polystyrene particles or particles with anionic surface modifications.^[^ ^100^ ^]^ Lastly, cationic PLGA particles formulated with PEI and CpG‐containing plasmid DNA did not induce CD83 upregulation in human DCs, while CD83 was upregulated with anionic PLGA particles administration or with plasmid alone.^[^ ^106^ ^]^ However, a similar experiment using PLA instead of PLGA particles failed to activate DCs by inducing either CD83 or CD86.^[^ ^106^ ^]^ Therefore, charge alone may not be enough to activate APCs, and it is possible that the effects of charged particles can differ depending on the polymer that is used for fabrication. In fact, most of the early work concluding that cationic particles were more favorable for APC interactions was performed using nondegradable polystyrene particles and may not be directly applicable to biodegradable polymers.^[^ ^99^, ^100^, ^101^ ^]^

Polymeric particles incorporating polycations may contain the added benefit of increased antigen presentation through the “proton sponge” effect. Polycationic polymers like PEI have a substantial H^+^ ion buffering capacity which is thought to promote disruption of the endolysosome through increased Cl^−^ ion influx. Such disruption is thought to lead to cross‐presentation in multiple particle systems and enhanced major histocompatibility complex class I (MHC I) presentation.^[^ ^107^, ^108^, ^109^ ^]^ However, it should also be noted that a smaller range of particle surface charge magnitudes has been studied for adaptive immune responses. This is because antigen is not necessarily required for in vitro studies that only investigate particle interactions with APCs, and the adsorption of antigen to the particle reduces the magnitude of the particle surface charge.

The effect of charged particles on APC function carries over to APC priming of T‐cell responses. Following increased uptake and activation of APCs, injected cationic particle vaccines show increased T‐cell responses in mice compared with anionic particles.^[^ ^98^, ^110^ ^]^ Similarly, anionic particles of significant surface charge magnitude can also exhibit increased CD8 + T‐cell responses over neutral particles.^[^ ^103^ ^]^ However, a confounding factor for the observation of greater T‐cell response from cationic particles is the absence of endotoxin testing. Endotoxin is negatively charged and may adsorb better to positively charged particles. As endotoxin itself is a potent immune stimulator, a greater association of endotoxin with cationic particles than with anionic particles has the potential to exhibit a greater immune response for cationic particles. This ambiguity illuminates the importance of endotoxin detection to eliminate a confounding factor from the assessment of vaccine formulations.

Despite an effect on cellular responses, there seems to be minimal direct surface charge‐related effect on humoral responses. A wide range of surface charges on polymeric particle vaccines, both positively and negatively charged, have not achieved substantial differences in total antibody titers.^[^ ^98^, ^103^, ^110^ ^]^ Surface charge may impact the relative induction of different IgG subtypes, but it is unclear whether IgG1 or IgG2 subclasses are favored.^[^ ^98^, ^110^ ^]^ Indirectly, a greater magnitude of surface charge can allow greater adsorption of antigen to particles, leading to increased immune responses essentially via increased antigen dose.^[^ ^111^, ^112^ ^]^ Although the use of an adjuvant such as CpG can increase overall immune responses, the effects of adjuvant on immune response seem to be independent of effects due to surface charge.^[^ ^110^ ^]^

There are multiple instances in which anionic particles would be preferred over cationic particles as a delivery vehicle for injectable vaccines. For example, protein antigens can adsorb well to some anionic particles, potentially better than cationic particles depending on the isoelectric point (pI) of the specific protein.^[^ ^97^, ^112^, ^113^, ^114^, ^115^ ^]^ The use of anionic particles may be required for adsorption to proteins carrying positive surface charges at neutral pH. However, cationic particles are advantageous for vaccine delivery of RNA or DNA. Nucleic acids are negatively charged and adsorb well to cationic particles, and the cationic particles themselves may promote disruption of the endosomal membrane and facilitate release of the nucleic acid into the cytosol.^[^ ^111^, ^116^ ^]^ In addition, the targeting specificity of charged particles must be considered. All cells in the body generally have a negatively charged cell membrane which promotes attachment to positively charged particles. This property of cationic particles is commonly used to promote adherence and persistence of intranasal vaccines in the mucosal layer. However, cationic particles have the potential to adhere to cells nonspecifically, which may decrease their specificity for APCs. This disparity may be overcome by fabricating large‐enough particles that passively target phagocytes by requiring active phagocytosis or by active targeting through specific cell‐targeting motifs. As a final consideration, the nonspecific adherence of cationic particles may benefit persistence in tissue but impair particle persistence in circulation. Specifically, cationic particles are cleared faster than anionic particles after intravenous injection, probably due to a greater amount of opsonins binding to the particle as well as the particles binding to cell surfaces.^[^ ^117^ ^]^ For all these reasons, the specific system of antigen, adjuvant, injection method, and target for delivery must be carefully considered when incorporating materials that carry either positive or negative surface charge during the formulation of polymeric particle vaccines. Nonetheless, a charged particle system seems to enhance cellular immune responses to vaccines.

### 3.3 Particle Degradation

As mentioned earlier, a key feature of biodegradable polymeric particle vaccines is the depot effect, i.e., the sustained release of vaccine elements over time. In general, the rate of payload release from the particle depends on how quickly the particle degrades, as well as the rate at which the encapsulate can diffuse out of particle. Multiple factors, such as the polymer used, surface area, and hydrophobicity, may impact whether the particle degrades on the order of hours, days, or even months. One consideration for such particle vaccines is how the degradation rate lines up with the induction of immune responses. Innate immune responses, especially with the use of adjuvants, can be initiated within hours, whereas adaptive immune responses take several days to develop. Using pumps for delivery, investigators have shown that the distributing antigen doses over time, rather than a bolus dose, can also affect humoral responses^[^ ^118^ ^]^ and cellular responses.^[^ ^119^ ^]^ It may be possible to optimize the duration and intensity of the sustained vaccine element release from a particle system in a way that best supports immune response kinetics, resulting in increased vaccine effectiveness.

**Figure 3:**
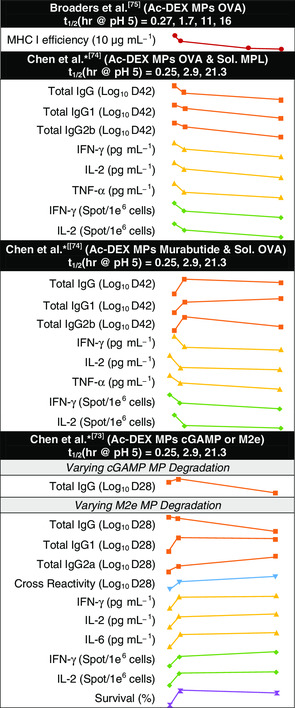
Summary of the effects of particle degradation on vaccine responses. A collection of sparkline plots designates the trend of indicated immunogenicity readouts for given degradation times. *t*_1/2_ is the time to half the degradation of the particles at pH 5, wherein Ac‐DEX is acid‐sensitive and degrades approximately two logs faster at pH 5 over pH 7. The t_1/2_ that corresponds to each point is given in the multi‐column black‐and‐white header for each antigen and/or adjuvant. The color of each plot represents a different readout: in vitro MHCI presentation (red), serum antibody generation (orange), antibody cross reactivity with other influenza virus strains (blue), antigen‐specific cytokine production with recall (yellow), functional T‐cell responses by ELISpot with recall (green), and survival after influenza viral challenge (purple). D indicates days. An * indicates that endotoxin was evaluated.

Exploiting the tunable degradation of acetalated dextran (Ac‐DEX), we have shown that release kinetics of vaccine elements play an important role in microparticulate vaccines. Most importantly, our studies indicate that antibody titers and cellular responses vary with degradation half‐lives (t_1/2_) for both adjuvant and antigen (**Figure** **3**).^[^ ^73^, ^74^ ^]^ Both Broaders et al.^[^ ^75^ ^]^ and Chen et al.^[^ ^74^ ^]^ illustrate that antibody and cellular responses using OVA were highest when using the fastest degrading material. However, when a smaller peptide antigen was used, such as the universal influenza matrix 2 protein ectodomain (M2e) which is 23 amino acids in length, the greatest response across immune readouts is with the slowest degrading material.^[^ ^73^ ^]^ Confocal imaging by Chen et al.^[^ ^74^ ^]^ illustrated that MP association with the phagosome differed between slow‐ and fast‐degrading particles, with fast‐degrading particles showing more delocalization within the cell. Although this early work supports that degradation rate plays a role in microparticulate vaccine trafficking, further work is needed to elucidate the exact mechanistic differences between the trafficking of slow‐ and fast‐degrading particles.

In addition, to degradation rate impacting antigen presentation, it seems to wholly affect adjuvant presentation in an independent manner. In two different studies, Chen et al.^[^ ^73^, ^74^ ^]^ varied degradation rate of particles encapsulating nucleotide‐binding oligomerization domain‐containing protein 2 (NOD‐2) agonist murabutide and the stimulator of interferon genes (STING) agonist cGAMP. With murabutide, the optimal humoral response was observed at the intermediate degradation rate, but the cellular responses peaked at the fastest degrading rate. For the cGAMP particles, the humoral response peaked at the intermediate degradation rate. The PRR for both of these studied agonists primarily resides in the cytosol.^[^ ^120^, ^121^, ^122^ ^]^ As such, different degradation kinetics may be optimal for adjuvants targeting receptors on the cell surface or in the endosome, such as TLR agonists. Though limited data makes general conclusions difficult, it is clear that degradation rate does play a role in adjuvant responses. This initial work also shows that degradation rate may differently influence antigen and adjuvant responses as well as differently influence cellular and humoral responses. Therefore, the degradation rate of polymers and other materials is another consideration for the design of vaccine delivery systems, as an optimal degradation rate may exist for each unique application.

### 3.4 Separate or Co‐Encapsulated Vaccine Elements

Although antigen and adjuvant play parallel roles in subunit vaccines to stimulate a potent vaccine response, their cellular processing pathways are not wholly dependent on one another. A typical protein antigen is internalized by APCs, processed and loaded onto MHC to be presented in a direct interaction with T‐cells. This is in sharp contrast to the adjuvant, which is not directly involved in the interactions between APCs and T‐cells. For example, R848 or other similar TLR agonists bind to an endosomal receptor. A cascade of pro‐inflammatory responses is then elicited from not only APCs but also other resident cells. It is not the adjuvant itself, but the downstream cytokines, chemokines, and costimulatory molecules that interact directly with T‐cells to stimulate an adaptive response. A system that delivers vaccine elements separately from one another would allow for more individual control over the cellular machineries that govern antigen processing and adjuvant signaling. This would allow individual optimization of vaccine element delivery, potentially leading to better vaccine responses.

**Figure 4:**
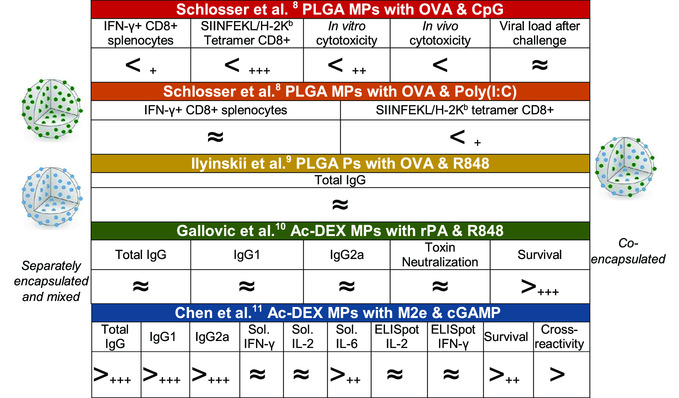
Summary of the effects of separate or co‐encapsulated vaccine elements on immune responses. Summary of publications where antigen (•) and adjuvant (•) are formulated in to separate (left‐hand side) or a single (right‐hand side) polymeric particle. A > sign indicates that separately encapsulated particles significantly outperformed co‐encapsulated for the aforementioned assay. Similarly, a < indicates that co‐encapsulated significantly outperform separately encapsulated. Sol. = soluble, as determined by ELISA after antigen restimulation. + *P*‐value < 0.05. ++ *P*‐value < 0.01. +++ *P*‐value < 0.001. A ≈ indicates that values were not noted to be significantly different from each other. Cross‐reactivity relates the average titer of sera antibodies to react to conscience M2e and M2e with 1, 2, and 3 changes in peptide sequence.

With conventional subunit vaccines, the idea of delivering antigen and adjuvant separately and at different times would likely require two injections. With polymeric particles, one injection can be used to deliver vaccine elements at different kinetics in a controlled fashion. Although there is a large body of literature that displays that co‐encapsulation of antigen and adjuvant or delivery of antigen adsorbed on adjuvant particles are effective in creating a protective immune response,^[^ ^123^, ^124^, ^125^, ^126^ ^]^ there have been limited studies which have explored the idea of co‐delivery versus separate (or mixed) delivery of vaccine elements using polymeric particle systems.^[^ ^73^, ^127^, ^128^, ^129^ ^]^ However, of the studies reported, PLGA and the tunable and acid‐sensitive biodegradable polymer Ac‐DEX^[^ ^130^ ^]^ have been used with mixed results (**Figure** **4**). To explore the individual role of antigen or adjuvant release kinetics, Chen et al.^[^ ^73^ ^]^ administered Ac‐DEX particle vaccines with antigen and adjuvant encapsulated in separate particles (Figure 4). Separate encapsulation revealed that an optimal degradation rate could be uniquely determined for each vaccine element that resulted in greater antibody, cellular, and protective responses. In fact, multiple reports show that antibody titers, cellular responses, and protective outcomes either are equivalent to or strongly favor separate encapsulation. In a model of influenza challenge, survival was significantly greater in the separately encapsulated group over the co‐encapsulated group when M2e was used with STING agonist cGAMP. This result was also observed when recombinant protective antigens (rPA) and R848 were used as part of an anthrax vaccine, wherein separate particles protected significantly better than co‐encapsulated particles.^[^ ^131^ ^]^

Although limited, the success of separate encapsulation seems contrary to the often‐accepted rationale for co‐encapsulation of vaccine elements within the same particle. Inclusion of both antigen and adjuvant within the same particle would ensure that both the “target” and “danger signal” are delivered to the same cell, particularly an APC. Therefore, both pro‐inflammatory activation and antigen presentation are promoted directly in individual APCs that take up the particles. This rationale is supported by Schlosser et al.,^[^ ^127^ ^]^ who showed cellular immune responses favoring co‐encapsulation over separate encapsulation when using PLGA MPs encapsulating OVA with either CpG or Poly(I:C). Despite greater cellular responses for co‐encapsulation over separate encapsulation in this system, viral load after challenge was not significantly different between encapsulation methods when using recombinant vaccinia virus expressing ovalbumin (rVV‐OVA). This limited data would indicate that some merit should be given to separately encapsulated vaccine elements, which may perform similarly or better than co‐encapsulated elements.

## 4 Considerations for Polymeric Particle Manufacturing

### 4.1 Scalability of Manufacturing Methods

A lesson learned from early PLGA tetanus vaccines is that scalability can impede the development of polymeric particle vaccines.^[^ ^53^ ^]^ This is underscored by the fact that cancer vaccines often do not require the same cost‐per‐dose benchmarks that are required for infectious disease vaccines, as application in resource‐limiting settings demands reduced costs.^[^ ^132^ ^]^ For example, the cost of manufacturing conventional vaccines in resource‐limited nations was reported as $0.98 to $4.85 per dose in 2019.^[^ ^133^ ^]^ For the RTS,S malaria vaccine that relies on a liposomal adjuvant (AS01), the cost is estimated at $5 per dose.^[^ ^134^ ^]^ To put this cost in perspective, the PLGA‐formulated antipsychotic risperidone (Risperdal Consta) costs $275 per dose commercially.^[^ ^135^ ^]^ With polymeric vaccine particle formulations, the increased costs emerge from the sterility requirement for vaccine manufacturing. After production in a very costly clean manufacturing environment, methods such as filtration, UV light, ethylene oxide, gamma‐irradiation, plasma, or autoclaving are used to sterilize these types of vaccines.^[^ ^136^ ^]^ Although filtration is the most conventional method, the filter size is on the order 0.2 μm and particles around or above this size can easily foul the filter, rendering it useless or too costly to continuously replace. Tangential flow filtration is often favored over perpendicular flow filtration, but it is a longer and more expensive process and can result in the degradation of particles that are susceptible to hydrolysis (e.g., polyesters). After filtration, the most reported preclinical method for polymeric particles is gamma‐irradiation for sterilization.^[^ ^137^, ^138^, ^139^ ^]^ All of these common sterilization methods can result in degradation of the polymeric integrity and thereby alter vaccine element release from the formulation.

One method to reduce manufacturing costs is to use a continuous rather than batch or semi‐continuous manufacturing process. A batch process is one that generates discrete amounts of product rather than a continuous output. For polymeric particle production, generation of emulsion particles in a glass beaker is considered a batch process, whereas generation of particles using a continuous flow impinger would be an example of a continuous process. With batch processes, the manufacturing cost can be significant because each post‐generation process (e.g., filtration) is staged for each batch and additional costs are associated with vessel cleaning, shut‐down, and up‐start to steady‐state procedures. In a continuous process, these interruptions can be mostly eliminated or significantly reduced. A semi‐continuous process is one which relies on one or more processes to be batch, whereas the remaining aspects of the process are continuous. Of the several manufacturing processes for particulate vaccines (**Table** **1**), many are batch at the preclinical scale, but can be continuous at the industrial scale.

**Table 1:** Manufacturing methods for polymeric nano/microparticles

Method	Description	Scalability	Typical solvents	Stirring involved?	pH	Size range reported	Temperature	Vaccines in vivo
Coacervation	Polymers in solution form a single colloidal coacervate solution facilitated through changes in pH.^[^ ^151^ ^]^	Continuous	Aqueous to organic solvent	Yes	Varies	μm	Ambient	Not widely reported.
Electrospray	Atomization of two liquid phases via electrostatic interactions to create a fine mist aerosol of droplets containing polymer and encapsulates.^[^ ^125^ ^]^	Continuous	Aqueous to organic solvent	No	Neutral	μm	Ambient	Anthrax^[^ ^129^ ^]^ Cytomegalovirus^[^ ^152^ ^]^ Ovalbumin^[^ ^74^ ^]^ Influenza^[^ ^137^ ^]^
Emulsion	Two immiscible liquid phases are mixed with stabilizers to form a colloidal suspension.^[^ ^153^ ^]^	Semi‐continuous	Aqueous and organic solvent	Yes	Neutral	nm–μm	Ambient	Widely reported. Influenza^[^ ^73^, ^85^, ^87^, ^93^, ^127^, ^128^, ^129^ ^]^ Albumin^[^ ^94^, ^95^ ^]^ Hepatitis B^[^ ^86^ ^]^ Ovalbumin^[^ ^85^, ^91^ ^]^ Anthrax^[^ ^131^ ^]^ Melioidosis^[^ ^154^ ^]^ Tetanus^[^ ^90^ ^]^
Flash nanoprecipitation	Two liquid phase inlets are introduced into a T, Y, or other confined impingement mixer and exit through a single outlet flow.^[^ ^155^ ^]^	Continuous	Aqueous to organic solvent	No	Neutral	nm–μm	Ambient	Not widely reported.
Ionic gelation process	Two liquid solutions form a gel due to ionic interactions.^[^ ^156^ ^]^	Continuous	Aqueous	Yes	Neutral	μm	Ambient	Diphtheria^[^ ^157^ ^]^ Influenza^[^ ^158^ ^]^
Layer‐by‐layer	Deposition of oppositely charged layers alternately.^[^ ^159^ ^]^	Batch	Aqueous to organic solvent	Sometimes	Varies	nm–μm	Ambient	Ovalbumin^[^ ^160^, ^161^ ^]^
Microfluidics	Microfluidic channels are formed to create mixing of miscible or immiscible liquid phases.^[^ ^162^ ^]^	Continuous	Aqueous to organic solvent	No	Neutral	nm–μm	Ambient	Not widely reported.
Polymerization	Reaction of monomers in solution with encapsulates to form polymeric particles.^[^ ^163^ ^]^	Batch	Aqueous to organic solvent	Yes	Varies	nm–μm	Varies	Ovalbumin^[^ ^84^, ^164^ ^]^
Particle replication in nonwetting templates (PRINT)	Micromolding soft lithography technique that uses a superhydrophobic mold to generate particles.^[^ ^165^ ^]^	Continuous	Aqueous to organic solvent	No	Neutral	nm–μm	Elevated	Dengue^[^ ^166^ ^]^ Influenza^[^ ^167^, ^168^ ^]^ Malaria^[^ ^167^ ^]^ Ovalbumin^[^ ^169^ ^]^ Streptococcus^[^ ^167^ ^]^
Salting out	Similar to an coacervation, except a salt is used to impede dissolving of organic phase into the aqueous phase.^[^ ^170^ ^]^	Semi‐continuous	Aqueous and organic solvent	Yes	Neutral	nm–μm	Ambient	Ovalbumin^[^ ^171^ ^]^
Spray drying	A liquid or slurry is rapidly dried with a hot gas phase.^[^ ^172^ ^]^	Continuous	Aqueous to organic solvent	No	Neutral	μm	Elevated	Anthrax^[^ ^143^ ^]^
Supercritical fluids	Supercritical fluids are used as a solvent or co‐solvent and undergo a pressure or temperature change to form particles.^[^ ^173^ ^]^	Continuous	Aqueous to organic solvent	Sometimes	Neutral	nm–μm	Reduced	Tetanus^[^ ^174^ ^]^

### 4.2 Marketability of Scaled‐Up Vaccines

In addition to the need for inexpensive production, the commercialization of a vaccine requires profitability to interest pharmaceutical companies and investors. Vaccines have relatively low profitability, with global sales historically less than 2% of global therapeutic drug sales.^[^ ^140^ ^]^ Currently, the most administered vaccines are for influenza, with 25 or more influenza vaccine products. Approximately 80 million doses are administered annually in the US,^[^ ^140^ ^]^ which include inactivated viruses (most seasonal influenza vaccines), subunit (FluBlok), and live‐attenuated (FluMist) vaccines. There are several barriers to the influenza vaccine market that are reflected in other vaccine markets. These barriers include the cost of domestic clinical trials, the development of production methods that adhere to current Good Manufacturing Practices (cGMPs), and that production must be demonstrated prior to an FDA‐approved manufacturing license.^[^ ^140^ ^]^ The upfront cost to generate a vaccine is significant, and may not be returned due to government‐mandated limits on the cost per dose. In 2005, the US government allowed cost per dose was $6.80 for influenza vaccines.^[^ ^140^ ^]^ Notably, this is higher than what would be acceptable for application in resource‐limited areas. Although manufacturers have been noted to develop vaccines for positive marketing, the costs associated with polymeric particle formulations make them less attractive compared with more conventional, cost‐effective vaccines. Therefore, polymeric particle vaccines must demonstrate transformative results to compel clinical translation. Such transformative results can include facile needle‐free application, decreased dosing frequency, significantly increased efficacy, reduced time to manufacture, and efficacy against a pathogen not otherwise achievable.

Currently, there is a worldwide demand for a coronavirus vaccine for the COVID‐19 pandemic. This has led to over 20 vaccine candidates in clinical trials and over 140 more in preclinical evaluation, made by almost as many developers.^[^ ^25^, ^141^ ^]^ Vaccines developed for a pandemic seem to be an exception for otherwise low profitability, as $3.3 billion in sales accompanied vaccines made in 2009 during the H1N1 influenza pandemic.^[^ ^142^ ^]^ A first‐to‐market vaccine can carry the advantage of gaining the largest contracts, which was seen with Novartis’ H1N1 influenza vaccine in the US during the 2009 pandemic. However, a pandemic can also serve as an opportunity for new classes of vaccines to be accepted into the market. Though the FluMist vaccine (Astra Zeneca) did not have the highest revenue of the four H1N1 vaccines, the otherwise unpopular intranasal delivery of an influenza vaccine was showcased during the pandemic. FluMist popularity was consequently increased for seasonal influenza vaccines in subsequent years.^[^ ^142^ ^]^ The current pandemic environment may serve as a similar opportunity for mRNA vaccines to gain market popularity, as several mRNA vaccines are in clinical trials for COVID‐19 and seeking FDA and international regulatory approval, with many more are in preclinical development. Several particle‐based vaccines for COVID‐19, such as lipid complexes and VLPs, are also in development.^[^ ^25^, ^141^ ^]^ Whether the current pandemic environment will garner advances for polymeric particle vaccines is still yet to be determined.

### 4.3 Effect of Manufacturing Methods on Vaccine Efficacy

Considerations already required for the manufacturing of more conventional subunit vaccines also apply to polymeric particle vaccines, with some additional ones. Similar to most vaccines, efficacy is decreased if protein or peptide contained within the formulation is denatured. Tertiary and even quaternary protein structures are often needed to generate neutralizing antibody responses. Evaluation of protein denaturation in particle vaccine formulations can be achieved through various methods, including circular dichroism, monoclonal antibody binding, and FTIR.^[^ ^129^, ^143^ ^]^ Although manufacturing parameters such as temperature, pH, exposure to solvents, and stirring speeds may impact protein/peptide denaturation and potentially decrease vaccine efficacy, the effect of these parameters during manufacturing is not well‐known due to a limited amount of studies. For example, several studies have noted the benefit of polymeric encapsulation in enabling storage outside the cold chain.^[^ ^143^, ^144^ ^]^ However, these vaccine efficacy studies primarily focus on the effect of the final product storage temperature and not on the effect of the manufacturing temperature. Similarly, the effect of pH is not well‐studied, though known to be involved in protein denaturation. High‐speed stirring or mixing during manufacturing can have an especially large impact on easily denatured proteins, such as the anthrax PA. In a mouse model using Ac‐DEX MPs encapsulating recombinant PA, both neutralizing antibody titer and protective efficacy were significantly reduced with emulsion particles formed using high‐speeding stirring compared with particles formed with low‐shear production through electrospray.^[^ ^129^, ^145^ ^]^ A manufacturing parameter more common to polymeric particle vaccines than to other subunit vaccines is exposure to solvents, though the effects can often be mitigated by solvent choice. A good basis for solvent choice is the FDA classification for residual solvents in therapeutics. Class 3 solvents (e.g., acetone, butanol, ethanol, ethyl acetate) are generally allowable without justification at doses of up to 50 mg per day. Class 2 solvents (e.g., acetonitrile, methanol, chloroform, hexane, dichloromethane) are evaluated at a case‐by‐case basis, and Class 1 solvents (e.g., benzene) should not be used.^[^ ^146^ ^]^ The use of Class 2 and Class 1 solvents can submit proteins to harsh environmental conditions during manufacture and should therefore be limited. Limiting the duration of protein exposure to solvents can also mitigate protein denaturation. Overall, there are a significant number of factors to consider when developing new methods for generation of polymeric formulations as well as applying or modifying existing methods. Further advances in manufacturing techniques that preserve protein structure may aid polymeric particle vaccines in achieving a marked advantage over other vaccines and compel their commercialization.

## 5 Conclusion

The past several decades have yielded advances that may one day transform the field of polymeric particle fabrication for infectious disease vaccines. Particle size has been shown to have an impact on the immune response, but each formulation is seemingly unique, such that broad all‐encompassing conclusions regarding cellular or humoral responses regarding size cannot be made. Imparting surface charge onto the particle seems to benefit at least cellular immune responses, though whether a negative or positive charge is favored seems to depend on the polymer and antigen used. The rate of particle degradation has been shown to influence cellular and humoral responses, varying for each antigen and adjuvant, which supports evidence that encapsulation of antigen and adjuvant separately in different particles has increased immunogenicity and efficacy than co‐encapsulated formulations. Further study into the immuno‐pharmaceutics surrounding this effect is warranted. For best translation to the clinic, all these design parameters must be incorporated into a manufacturing process that is continuous, affordable, and does not denature the protein antigen. Given that each of these considerations vary with the type of polymer used and method of fabrication, it stands to reason that characterization is required for each individual formulation.

As the now mature field of micro/NPs for vaccines continues forward, there are several established and nascent biomaterial platforms that are being applied to vaccines. Many of these platforms rely on microfabrication techniques^[^ ^147^ ^]^ such as micro/nanoneedles,^[^ ^148^ ^]^ micro‐electro‐mechanical systems (MEMS),^[^ ^149^ ^]^ and reservoir microdevices.^[^ ^150^ ^]^ Incorporation of an established method such as microfabrication provides some level of manufacturing ease over particle formation; however, a clean environment during manufacturing is still necessary and at significant cost. Studies are just beginning on several techniques to feasibly manufacture polymeric particles for infectious disease vaccines. As the research on improving manufacturing techniques continues, clinical translation is certain to follow.

## Conflict of Interest

The authors declare no conflict of interest.
